# On abstraction: decoupling conceptual concreteness and categorical specificity

**DOI:** 10.1007/s10339-020-00965-9

**Published:** 2020-03-16

**Authors:** Marianna Bolognesi, Christian Burgers, Tommaso Caselli

**Affiliations:** 1 Faculty of Modern Languages and Cultures, University of Bologna, Via Cartoleria 5, 40124 Bologna, Italy; 2 Department of Communication Science, Vrije Universiteit Amsterdam, De Boelelaan 1081, 1081 HV Amsterdam, The Netherlands; 3 Amsterdam School of Communication Research (ASCoR), University of Amsterdam, Nieuwe Achtergracht 166, 1018 WV Amsterdam, The Netherlands; 4 Faculty of Arts - Center for Language and Cognition Groningen, Rijksuniversiteit Groningen, Oude Kijk in’t Jaatstraat 26, 9712 CR Groningen, The Netherlands

**Keywords:** Abstraction, Abstract concepts, Concrete concepts, Generic categories, Specific categories

## Abstract

Conceptual concreteness and categorical specificity are two continuous variables that allow distinguishing, for example, *justice* (low concreteness) from *banana* (high concreteness) and *furniture* (low specificity) from *rocking chair* (high specificity). The relation between these two variables is unclear, with some scholars suggesting that they might be highly correlated. In this study, we operationalize both variables and conduct a series of analyses on a sample of > 13,000 nouns, to investigate the relationship between them. Concreteness is operationalized by means of concreteness ratings, and specificity is operationalized as the relative position of the words in the WordNet taxonomy, which proxies this variable in the hypernym semantic relation. Findings from our studies show only a moderate correlation between concreteness and specificity. Moreover, the intersection of the two variables generates four groups of words that seem to denote qualitatively different types of concepts, which are, respectively, highly specific and highly concrete (typical concrete concepts denoting individual nouns), highly specific and highly abstract (among them many words denoting human-born creation and concepts within the social reality domains), highly generic and highly concrete (among which many mass nouns, or uncountable nouns), and highly generic and highly abstract (typical abstract concepts which are likely to be loaded with affective information, as suggested by previous literature). These results suggest that future studies should consider concreteness and specificity as two distinct dimensions of the general phenomenon called abstraction.

## Introduction

Consider your kitchen table. This table is an instance of the category^1^ TABLE, which we label with the word *table*. TABLE is commonly classified as a concrete concept, because the specific instances that constitute the category TABLE (including your kitchen table) can be perceived through sensory experiences: Tables can be seen, touched, and smelled, among other things.

Perceptibility (Connell and Lynott 2012) is arguably one of the core dimensions of meaning that allow us to discriminate what is *concrete* from what is *abstract*: TABLE is a concrete concept because the instances that constitute this conceptual category and form the related concept are directly characterized by features that describe (also) perceptual properties. Conversely, THEORY is abstract because instantiations that can be labeled with the word *theory* are not directly characterized by perceptual properties: An instance of theory, or simply, *a* theory, cannot be directly experienced through any of the senses (sight, touch, smell, among others).^2^

Consider now FURNITURE. The category FURNITURE encompasses subcategories such as TABLE (and more specific types of tables), as well as individual instances of tables like your specific kitchen table. A core difference between the two categories, FURNITURE and TABLE, is that the first is more generic than the latter. FURNITURE is not characterized *directly* by perceptual features because it is an encompassing category that includes a variety of subcategories (TABLES, COUCHES, CHAIRS, etc.) which, in turn, can be characterized by perceptual features (e.g., TABLE has four legs, can be made of wood, etc.; COUCHES are soft, etc.). As a conceptual category, FURNITURE is therefore more detached and more distant from fleshy perceptual features than TABLE. Moreover, FURNITURE is overall less rich of features than TABLE, because the latter is a basic-level category, while the former is a generic category (Berlin 1973; Rosch 1975; Rosch et al. 1976).

Both FURNITURE (generic) and THEORY (abstract) are conceptual categories that lack direct perceptual features, and are, in this sense, different from categories such as TABLE (which is concrete and specific).

This paper investigates the relation between Specificity, defined as the gradable property that characterizes concepts on a scale from highly specific (e.g., concepts referring to individuals) to highly generic (e.g., concepts referring to groups or classes), and Concreteness, defined as the gradable property that distinguishes concepts on a scale from being characterized by lots of sensory-based information (i.e., highly concrete) to virtually none of it (i.e., highly abstract).^3^ Based on these observations, it could be deduced that generic categories (e.g., FURNITURE) are perceived to be more abstract than specific ones, because they are not directly defined by perceptual features. The aim of our study is to understand and to investigate how Specificity interfaces with Concreteness.

In particular, we formulated the following research questions:

- RQ1: Are *generic* concepts perceived to be more abstract than *specific* ones?
- RQ2: To which extent does the distinction between abstract and concrete entities in the WordNet (WN, henceforth) taxonomy (Fellbaum 1998) reflect the concreteness perceived by speakers? And if the WN-based distinction between abstract and concrete is backed up by participants’ judgments, do the words within these two WN branches display statistically significant differences in their average degree of specificity?
- RQ3: What type of concepts can be found in the four intersections of the two variables (Specificity and Concreteness)?

Through a series of quantitative and qualitative analyses, we explore the relationship between Specificity and Concreteness and discuss the theoretical implications for a coherent theory of meaning that aims at decoupling the notions of concreteness and specificity.

The remainder of this paper is structured as follows: In the “Theoretical background” Section, we provide an overview of various definitions of the polysemous term *abstraction*, and show that two potentially different phenomena are included and, at least in some cases, conflated within its general definition: These are conceptual concreteness and categorical specificity. In the “Study 1” and “Study 2” sections, we report two quantitative studies in which we operationalize and compare Specificity and Concreteness for a large sample of nouns. In the “Study 3” section, we report on a qualitative analysis in which we cross the two variables and describe which type of words appears in the four quadrants (highly abstract and generic words, highly abstract and specific words, highly concrete and generic words, and highly concrete and specific words). Finally, in the “General discussion,” we overview the results of our investigations and its implications within the cognitive-science community.^4^

## Theoretical background

*Abstraction* is a cognitive phenomenon investigated in different disciplines (see Burgoon et al. 2013 for an overview) and defined in slightly different ways. In cognitive science, it is typically defined as a conceptual categorization process by which “knowledge of a specific category has been abstracted out of the buzzing and blooming confusion of experience” (Barsalou 2003: 389).

Barsalou (ibid.) distinguishes six senses of abstraction, namely:

1. Categorical knowledge (defined as the knowledge of a category being abstracted from experience);
2. Behavioral ability to generalize across category members (defined as the properties of a category’s members being summarized behaviorally);
3. Summary representation (defined as the controversial cognitive bases of the behavioral abstractions provided in sense 2);
4. Schematic representation (defined as the controversial sparse representations that represent categories in memory);
5. Flexible representation (defined as the controversial idea that summary representations can be applied flexibly to many different tasks);
6. Abstract concepts (“when concepts become detached from physical entities and more associated with mental events, they become increasingly abstract” Barsalou 2003: 1178).

In this complex and highly granular definition of abstraction, abstract concepts are characterized as *a type of* abstraction from physical entities (thus suggesting a definition in line with the categorical abstraction) that characterizes concepts more associated with mental events (thus suggesting a definition in line with conceptual abstractness). However, because it is a process that starts from experience, abstraction involves both abstract and concrete concepts alike: We abstract from experience to construct concrete conceptual categories (e.g., TABLE), and we abstract from experience to construct abstract conceptual categories (e.g., THEORY). Yet, the process of abstraction may work in different ways for abstract and concrete concepts, given that the differences between these two types of concepts are backed up by several empirical studies, showing that words designating concrete concepts are more easily processed than words designating abstract ones in a variety of tasks, including word recognition (e.g., Strain et al. 1995), memory tasks (Jefferies et al. 2006; Romani et al. 2008), comprehension tasks (Kounios and Holcomb 1994; Schwanenflugel and Shoben 1983), and production tasks (Goetz et al. 2007; Tyler et al. 2000; Wiemer-Hastings and Xu 2005). Moreover, in language development, words referring to abstract concepts tend to appear later in children’s vocabulary, compared to words that refer to concrete concepts (e.g., Vigliocco et al. 2017), and neuroscientific evidence shows that the two types of concepts can be specifically impaired in brain-damaged patients, because their processing relies on overlapping but partly distinct neural systems (e.g., Binder et al. 2009; Hoffman 2016 for literature reviews).

In general, the debate on category learning and representation has a very long history that taps directly onto the question of whether people represent categories in terms of an abstracted summaries, that is, prototypes (e.g., Posner and Keele 1968; Reed 1972; Rosch 1978; Smith and Minda 1998) or as sets of specific instances (e.g., Brooks 1978; Estes 1986; Medin and Schaffer 1978; Nosofsky 1986). The two views critically differ in terms of informativeness and economy (Komatsu 1992). Prototype-based representations depend on maximal abstraction, and therefore have an appealing economy but they fail to provide the detailed information people actually use. On the contrary, exemplar-based representations are established on minimal abstraction, and are therefore more informative and detailed, but not economical.

In a recent study on abstraction, Reed (2016) proposes a taxonomy of abstraction based on three senses of this term: *abstract* as the opposite of *concrete* (concept with no material referent); *abstract* as the opposite of *equivalent* (abstract entity that includes only some attributes of multicomponent stimuli); and *abstract* as the opposite of *particular* (abstract entity that applies to many particular instances of a category, like a category prototype). These three senses of abstraction, according to Reed (2016), refer to different granularities, where the first applies to instances, the second to the attributes of instances, and the third to categories of instances.

*Concreteness*, instead, is a variable that measures the degree to which a referent in the real world is associated with a specific concept that can be perceived through our sensory experiences. For example, a table can be perceived through our senses easier than a theory, and therefore the concept TABLE is more concrete than the concept THEORY. Most published studies operationalize concreteness in terms of perceptibility, which is measured by means of human-generated ratings. These can be expressed as comprehensive scores of concreteness (i.e., how concrete is TABLE on a Likert scale, e.g., Brysbaert et al. 2014) or as scores expressing the degree by which a concept can be perceived through each of the five senses—sight, touch, hearing, smell, taste (i.e., to what extent can TABLE be perceived, respectively, through sight, touch, etc., on dedicated Likert scales, as in Lynott and Connell 2013).

Although Abstraction and Concreteness are theoretically distinct, one describing the construction of conceptual categories starting from experiences, and the other describing the perceptibility of the referents designated by given concepts, the polysemous nature of the term *abstraction* in some cases may suggest that the two variables are conflated. For example, Burgoon et al. (2013) provide an extensive overview of the different ways in which abstraction has been defined in the literature and then suggest their integrative definition of abstraction as “a process of identifying a set of invariant central characteristics of a thing” (Burgoon et al. 2013: 502). They then propose that abstraction operates on a continuum, in which:

lower levels of abstraction (i.e., higher levels of concreteness) capture thoughts that are more specific, detailed, vivid, and imageable (e.g., Strack et al. 1985), often encompassing readily observable characteristics (e.g., furry dog, ceramic cup; Medin and Ortony 1989). Higher levels of abstraction (i.e., lower levels of concreteness), on the other hand, include fewer readily observable characteristics and therefore capture thoughts that are less imageable (e.g., friendly dog, beautiful cup). (Burgoon et al. 2013: 503).

In this integrative definition, the two variables that we described above are conflated.

The APA Dictionary of Psychology (VandenBoss 2006: 4) provides three different definitions of abstraction.

1. The formation of general ideas or concepts, such as “fish” or “hypocrisy,” from particular instances.
2. Such a concept, especially a wholly intangible one, such as “goodness” or “beauty.”^5^

On these, the first two suggest that the same label may be applicable to both Abstraction and Concreteness as they have been described above. While definition 1 seems to refer to the process of categorization, a phenomenon that we operationalize through a variable that we call Specificity, definition 2 refers to the perceptibility of the referent associated with a concept, a phenomenon that we operationalize through a variable that we call Concreteness. Both these variables are continuous, rather than binary, and while Specificity is a relational property that characterizes how generic/specific a category is, compared to other categories, Concreteness is an absolute property that characterizes how perceptible the referent denoted by a concept is.

Finally, in a recent attempt to clarify the relation between these two variables, Borghi and Binkofski (2014: 3) suggest that “concepts as “animal” and “furniture” (on top of the abstraction hierarchy) are more abstract than “dog” and “chair,” but their category members are all concrete instances.” In this series of studies, we investigate the relation between the two variables: Specificity (which operationalizes the process of categorical abstraction) and Concreteness (which operationalizes the perceptibility of a referent associated with a concept). We hypothesize that categories which are low in specificity (i.e., generic categories such as FURNITURE) are by definition more inclusive (Rosch 1975) and therefore less rich in defining features, specifically in perceptual features. Being low in perceptual features, such generic concepts might also be less tangible, or less concrete, and therefore more abstract.

In Study 1, we investigate whether *generic* concepts are perceived to be more abstract than *specific* concepts, based on an analysis of a sample of 13,518 English nouns. In particular, we correlate measures of specificity based on WordNet (WN), with concreteness ratings elicited from participants in experimental settings. The measures of categorical specificity are extracted from WN (Fellbaum 1998), an electronic database that encompass various semantic relations between word senses, by using the hierarchical hypernym relation characterizing the noun taxonomy. As Miller (1998) pointed out, such hierarchical organization of the nominal lexicon, despite a lack of good theoretical explanations, captures valid linguistic facts. The way in which this semantic relation is formalized is actually a good proxy to operationalize Specificity. For the variable Concreteness, we relied on a well-known database of concreteness ratings judgments collected in experimental setting by asking people to rate the concreteness of given words (Brysbaert et al. 2014).

In Study 2, we build on the results of Study 1 and recast our analysis: Using the explicit distinction made in WN between abstract and concrete entities, we test whether such distinction generates two samples of concepts that are significantly different from one another in terms of concreteness scores (Brysbaert et al. 2014) and in terms of specificity (using our measures extracted from WN).

Finally, in Study 3, we cross specificity and concreteness, and provide a qualitative analysis of the types of nouns that appear to be highly specific and highly concrete, highly specific and highly abstract, highly generic and highly concrete, and highly generic and highly abstract.

## Study 1

### Method

WN is a large lexical database of English words created in the Cognitive Science Laboratory of Princeton University in 1985. Entries cover the major parts-of-speech, such as verbs, nouns, adjectives and adverbs, and are organized via sets of cognitive synonyms, called *synsets*. Each synset represents a distinct concept—or, as stated by Miller (1998), an instance of a lexicalized concept—and is inter-linked to other synsets through lexical and conceptual-semantic relations.

Entries covering nouns in WN are primarily structured by two main semantic relations: (1) *synonymy*, and (2) *hypernymy*/*hyponymy*, or subsumption/subordination. The latter relation links more generic concepts to more specific ones (e.g., FURNITURE is a hypernym of a TABLE). The *hypernymy*/*hyponymy* relation, usually abbreviated as IS-A within WN-based studies (e.g., DOG IS-A MAMMAL), is hierarchical, asymmetric and transitive: All properties of superordinate elements are directly inherited by their subordinate nodes. It shall be observed that it is incorrect to consider the WN noun hierarchy as a single taxonomy, because, in various versions of WN, there are multiple top root nodes, which give birth to multiple hierarchies. In WN 1.5 (released in 1995), for instance, there are 11 unique top nodes, or unique beginners (Miller 1998), covering distinct conceptual and lexical domains. In this work, we used the most recent version of WN, i.e., WN 3.0, as available in the Natural Language Toolkit (NLTK) Python library (Bird et al. 2009).^6^ In this version, the top node ENTITY has three direct hyponyms: ABSTRACTION, defined as “a general concept formed by extracting common features from specific examples” (abstraction.n.06), PHYSICAL_ENTITY, corresponding to “an entity that has physical existence” (physical_entity.n.01), and THING, whose gloss is “an entity that is not named specifically” (thing.n.08). To address our first question, whether *generic* concepts are perceived to be more abstract than *specific* ones, we first established how specificity measures can be extracted from WN.

To calculate specificity scores, we relied on WN’s IS-A relation^7^ of the synset words, i.e., the words composing each synset. The basic idea motivating how we formalized our measure of specificity is the following: If we imagine WN as an upside-down tree, in which the top root nodes constitute the most generic concepts, and the nodes at the very bottom of the tree (or wherever a branch ends), i.e., the leaves, constitute the most specific concepts, then the relative position of a concept within the tree (i.e., the number of nodes to the top root and the average number of nodes from the concept to each of the leaves) provides a good proxy, i.e., indirect evidence, of how specific a concept is, compared to all the other concepts represented in WN.

#### Specificity measures

Lexical resources that explicitly contain information about specificity as defined in this paper are not existent. Previous work in various branches of linguistics, including computational linguistics, have focused on the interpretation of referents of nouns/noun phrases as individuals (specific) or kinds (generic) (Krifka et al. 1995; Mitchell et al. 2003; Poesio 2004; Reiter and Frank 2010; Louis and Nenkova 2011; Friedrich et al. 2015, among others). Only in a very recent empirical study, Iliev and Axelrod (2017) address this issue. They proposed two measures to determine the abstractness—or better, the genericity—of a noun based on its position in the WN hierarchical taxonomy. Iliev and Axelrod (2017) distinguished two measures, one called *precision*, which determines the distance of a word from the top root of the taxonomy (e.g., the number of nodes between TABLE and ENTITY) and one called *inclusiveness*, which determines the distance of a lemma from its bottom leaves. This second measure quantifies the “amount of information associated with a concept. The more precise a concept is, the more information it provides, since all the information contained in a parent node is inherited by a child node, but the opposite is not true” (Iliev and Axelrod 2017: 717).

**Fig. 1:**
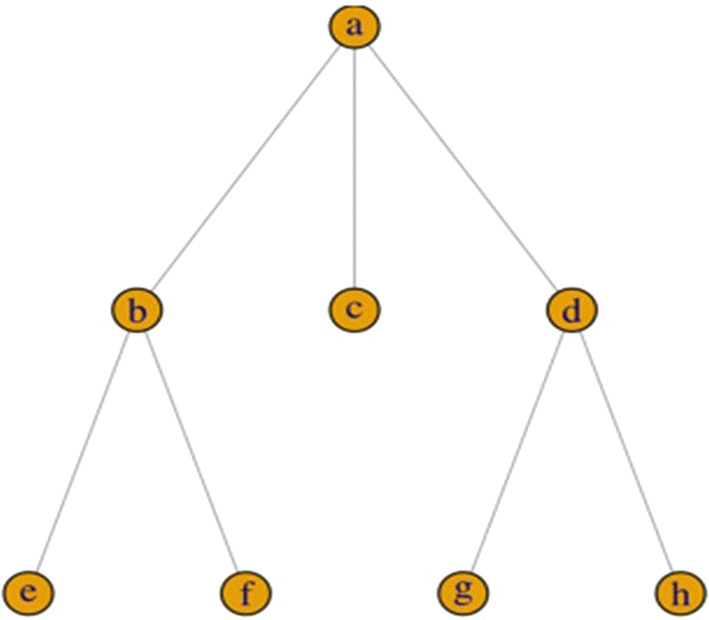
Extracted from Iliev and Axelrod (2017: 720). A virtual excerpt of the WN taxonomy

Figure 1 shows a virtual excerpt of the WN taxonomy. Lemmas *C* and *E* have the same degree of *inclusiveness* (i.e., distance from leaves = 0) but different degrees of precision (i.e., different numbers of nodes to the top root). Both measures contribute to determine the relative position of C and E in the taxonomy, and both carry information about their overall degree of specificity (intended as a combination of number of hypernyms and number of hyponyms).

For the purpose of our study, we wanted to determine the relative position of a word in the WN taxonomy with one measure, in order to make specificity comparable with concreteness (which is also expressed by one measure). We therefore need to combine what Iliev and Axelrod (2017) call *precision* and *inclusiveness* into a single measure. To achieve this goal, we designed three measures, which we describe and motivate below. A crucial aspect that led us to use three measures (instead of one) is the following. Specificity, as previously mentioned, is a relational property: A category is more or is less specific, in relation to another category. Conversely, concreteness can be measured in absolute terms, as the perceptibility of the referent. Being a relational property, the specificity of a category must be measured by taking into account the specificity of other categories, within the taxonomy of category. However, from the perspective of cognitive plausibility we are unable to determine how much of the information provided by the categories in a taxonomy contributes to determine the specificity of one category. For this reason, we opted for three measures of specificity, each operationalizing the amount of information recruited from the taxonomy in different ways.

Our first proposed measure of specificity, based on a previous measure proposed by Resnik (1995), operationalizes the specificity of a word by counting the number of nodes between a word and its leaves, added to the number of nodes between that word and the top root. We call this measure **Specificity 1**, and we formalize it as follows:

$${\text{Specificity}}\;1 = d + \log \left( {\left( {1 + n} \right)/N} \right)$$

where *d* is the number of nodes between a word and the top root node, *N* is the total number of nodes in the WN taxonomy restricted to nouns (i.e., 82,115), and *n* is the total amount of direct and indirect hyponyms of a given word. Note that *n* is increased by 1 to account for the node itself, and to avoid division by zero in case of final leaf nodes (i.e., nodes that do not have any hyponyms).

In case of polysemous entries, such as for the word “dog” that is associated with multiple synsets, e.g., “domestic animal” (dog.n.01 in WN), “frump” (dog.n.02 in WN), we decided to take always the first sense, or concept. We opted for this solution because most of the concepts in WN 3.0 are ordered using sparse data from semantically tagged texts (e.g., the SemCor Corpus), resulting in the most common uses of a concept (or, in other words, the most common senses associated with a synset word) listed above others.^8^ Whenever a word presents multiple hypernyms (a phenomenon called **multiple inheritance**, which we describe and problematize in our second study), this measure follows all available paths up to the root node.^9^

Our second measure, **Specificity 2**, is a variation of Specificity 1, where the value of *N* in the original Resnik (1995) formula is not the total number of nodes within WN, but instead it is restricted to the total number of nodes in the two taxonomy branches displaying ABSTRACTION (abstraction.n.06) or PHYSICAL_ENTITY (physical_entity.n.01) as top root nodes. For instance, for “dog,” whose first sense, dog.n.01, ends at PHYSICAL_ENTITY, the value of *N* at the denominator is the total number of nodes in the taxonomy, i.e., synsets that have as a top node PHYSICAL_ENTITY, i.e., 39,555. On the other hand, for “inception,” whose first sense, inception.n.01, ends at ABSTRACTION, the value of *N* at the denominator is 38,668.

Finally, we operationalized one additional measure of specificity, in which we aimed at simplifying the calculations as much as possible, taking into account the overall total depth of WN, instead of the number of hyponyms of each word. **Specificity 3** returns the specificity score of each entry by dividing the amount of direct and indirect hypernyms of a target word by the maximum depth of the WN noun taxonomy, i.e., the maximum distance from the ENTITY root node to a leaf. The formula for Specificity 3 is:

$${\text{Specificity}}\;3 = \left( {1 + d} \right)/20$$

where *d* is the total amount of direct and indirect hypernyms of a target word and 20 is the maximum distance (i.e., depth) of a synset word from the ENTITY top node. Note that dividing *d* by the maximum length of the noun taxonomy enables us to compare nodes in different paths of the taxonomy and, most importantly, to compensate for the possibility that all leaf nodes may result in having the same Specificity score regardless of their actual position in the taxonomy.^10^

#### Materials

The database of concreteness ratings collected by Brysbaert and colleagues encompasses 15,030 lemmas labeled as nouns. Of these, only 13,518 can be retrieved in WN using the three specificity measures, and worked as a basis for our studies. In particular, they were all used for Study 1. The remaining roughly 2 K either are not attested in WN, or cannot be properly weighted using our measures of specificity.

#### Procedure

For the 13,518 WN nouns for which a concreteness score was available in the database by Brysbaert et al. (2014), we calculated the degree of specificity, using the three different measures described above. The specificity scores were then normalized into a 5-points scale, to better compare them with Brysbaert’s concreteness scores, which have been measured on 5-points Likert scales.

**Fig. 2:**
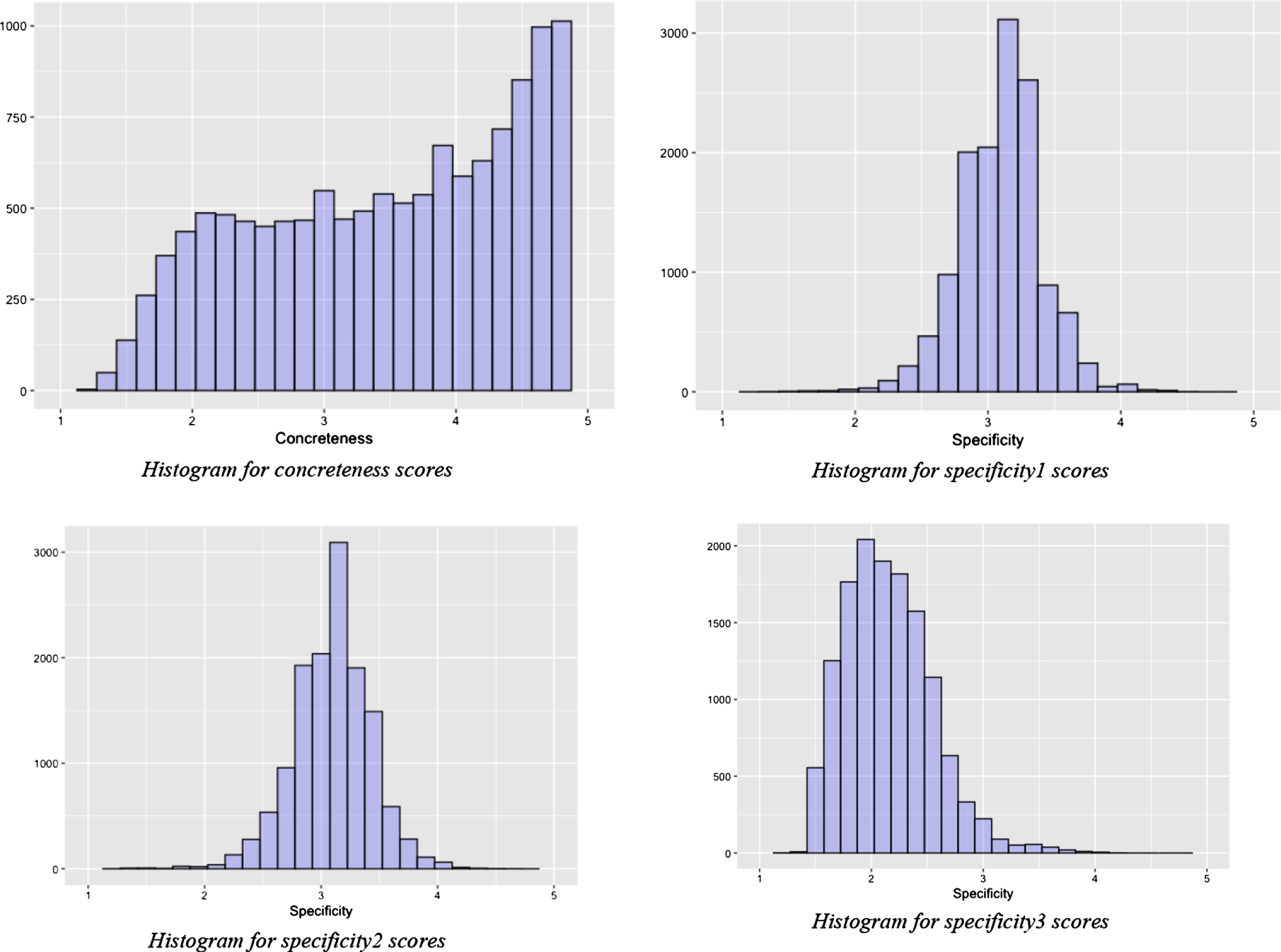
Histograms of concreteness, specificity 1, specificity 2, and specificity 3 (Study 1)

Next, we checked whether the data were normally distributed. Both a visual inspection of the histograms (see Fig. 2) and QQ plots, and indications of skewness (concreteness: − 0.338; specificity 1: − 0.188; specificity 2: − 0.325; specificity 3: 0.747) and kurtosis (concreteness: 1.867; specificity 1: 4.400 specificity 2: 4.493; specificity 3: 4.182) indicated that the normality assumptions were violated in all four variables. The high kurtosis values indicate that our variables have heavy tails. In such situations, Spearman’s correlation coefficients are preferred over traditional Pearson correlation coefficients (de Winter et al. 2016). To facilitate comparison with other studies, we also present Pearson’s correlation coefficients.

We thus subsequently calculated the correlation between each of the three specificity measures illustrated in “Specificity measures” section and the concreteness judgments. This analysis demonstrates the degree to which each specificity measure on its own is related to concreteness, and shows the degree of overlap between the two dimensions of abstraction.

### Results

Table 1 reports the average concreteness and the average specificity measures for the sample of 13,518 nouns analyzed, and the correlations between the concreteness scores and the three measures of specificity.

**Table 1:** Average concreteness and specificity scores for the 13 K nouns analyzed and Pearson’s and Spearman’s correlation coefficients between the different specificity measures and the concreteness scores

	Concreteness (Brysbaert et al. 2014)	Specificity 1 (WN)	Specificity 2 (WN)	Specificity 3 (WN)
*N* = 13,518	*M* = 3.558 SD = 1.019	*M* = 3.103 SD = 0.311	*M* = 3.092 SD = 0.332	*M* = 2.192 SD = 0.378
Pearson’s/Spearman’s correlation coefficients with the Brysbaert et al. (2014) concreteness scores	–	*r* = 0.263/0.272, *p* < .001	*r* = 0.267/0.295, *p* < .001	*r* = 0.354/0.361, *p* < .001
Pearson’s/Spearman’s correlation coefficients with the Specificity 1 (WN) scores	–	–	*r* = 0.988/0.991, *p* < .001	*r* = 0.847/0.858, *p* < .001
Pearson’s/Spearman’s correlation coefficients with the Specificity 2 (WN) scores	–	–	–	*r* = 0.822/0.842, *p* < .001

*WN* Word Net

As Table 1 shows, the average concreteness of the 13 K nouns is roughly in the middle of the 5-points Likert scale of the database, slightly skewed toward high concreteness. The average specificity obtained with the three measures is more varied: Specificity 1 and Specificity 2 are slightly more skewed toward the pole of specificity, while Specificity 3 is more skewed toward the pole of genericity, in relation to the 5-point scales on which such measures were normalized (1 being highly generic and 5 being highly specific, see also Fig. 2). The Pearson’s and Spearman’s correlation coefficients of each of the three specificity measures with the concreteness ratings are weak, although positive (Evans, 1996). However, when analyzing the correlations in terms of effect sizes (Cohen 1992), the results are differentiated as Specificity 1 and Specificity 2 have a small–medium effect, while Specificity 3 has a medium–high effect size.

Next, we wanted to explore whether the three specificity measures were comparable, or explained unique parts of the variance in the relation with concreteness ratings. However, Pearson correlation coefficients between the three specificity measures were very high (see Table 1). Due to these high correlation coefficients between the three different specificity measures, we could not estimate a reliable regression model that contained more than one of the specificity measures.^11^ This suggests that the three specificity measures may be rather comparable.

### Discussion

Our first general research question is whether there is a significant correlation between specificity and concreteness, and whether, in particular, generic concepts are perceived to be less concrete than specific ones.

Based on three measures of specificity, which we partially retrieved from the literature and partially constructed based on our research goals, we observed relatively low correlations between the variables of concreteness and specificity: The specificity of a concept (i.e., its hierarchical position within a lexical taxonomy) only has a relatively small relation to its perceived concreteness. The two phenomena seem to be mostly independent from one another, at least when we operationalize abstractness by means of scores of perceived concreteness by humans, and abstraction by means of hypernym relations encoded in WN.

However, some properties of WN might have influenced our results. WN treats some aspects related to the polysemy of the IS-A relations (Wierzbieka 1984, Miller 1998) in a peculiar way by attributing multiple hypernyms to the same word sense. Multiple inheritance is not a problem per sé as it is a way of addressing the polysemy of the hypernym relation. However, in some cases the multiple inheritance gives rise to a “tangled” taxonomy (Fahlman 1979) by connecting a concept to multiple hypernyms that feed into branches of WN that are not ontologically compatible. For instance, as reported in Verdezoto and Vieu (2011), in version 3.0 of WN, the first synset of LETTER (letter.n.01) inherits the physical aspect meaning from its hypernym “document” (document.n.02), which has PHYSICAL_ENTITY at the final top root node, *as well as* the abstract meaning (the content of a written letter), which links to the hypernym “text” (text.n.01), which in turn ends with the root node ABSTRACTION. The two higher hypernyms (PHYSICAL_ENTITY and ABSTRACTION) are not ontologically compatible for the same word sense (the first synset of LETTER). For the purpose of our investigation, which focuses on specificity as well as concreteness, words referring to concepts that are listed in WN as both abstract *and* concrete are problematic: They constitute a technical problem for calculating the specificity measures as well as a theoretical problem (concepts being both abstract and concrete). We took these peculiarities into account and recast our analyses in Study 2.

## Study 2

In this second study, we investigated whether the nouns that are listed either as abstract or as concrete in WN, that is, under the nodes ABSTRACTION or PHYSICAL_ENTITY, have significantly different degrees of concreteness and specificity.^12^ If the words listed under PHYSICAL_ENTITY prove to be significantly more concrete than those under ABSTRACTION (in terms of concreteness ratings elicited from speakers), then, according to our initial hypothesis, we would expect them to be also significantly more specific.

### Materials and procedure

To operationalize this question, we first compared the list of nouns used in Study 1 with those appearing within each of the two WN branches, and retained only those nouns that appeared under one of the two nodes, i.e., ABSTRACTION or PHYSICAL_ENTITY, removing any possible cases of words with multiple inheritance. This means that we excluded all cases of multiple inheritance appearing in ontologically compatible branches (e.g., FOOTMAN, footman.n.01, has two hypernyms: LIVING_ENTITY and CAUSAL_AGENT, both pointing to PHYSICAL_ENTITY) as well as multiple inheritance appearing in ontologically incompatible branches (e.g., BLOODSTREAM, bloodstream.n.01, has two hypernyms: MATTER, which ends into PHYSICAL_ENTITY, and PART, which ends into ABSTRACTION).

We then compared the concreteness scores using a Welch’s *t*-test (Delacre et al. 2017) associated with the nouns appearing in the two branches, ABSTRACTION or PHYSICAL_ENTITY, to test whether the WN-based distinction between abstract and concrete nouns was substantiated by psychological data (i.e., concreteness ratings).

Subsequently, we compared in a Welch’s t-test our measures of specificity, to test whether words that have PHYSICAL_ENTITY as a hypernym (and therefore are listed as concrete in WN) are also significantly more specific than words that have ABSTRACTION as hypernym.

### Results

Of the 13,518 nouns analyzed in study 1, a total of 3896 nouns are listed in WN under the node ABSTRACTION and 3146 under the node PHYSICAL_ENTITY. The number of words within one of the two branches and without multiple inheritances adds up to 7042. Table 2 shows the average concreteness scores associated with the words in these two samples and the results of the two Welch’s *t*-tests.

**Table 2:** Average concreteness of the words appearing under the PHYSICAL_ENTITY and under the ABSTRACTION nodes in WN

	Concreteness (Brysbaert et al. 2014)	Specificity 1	Specificity 2	Specificity 3
All words (ABSTRACT + CONCRETE) (*n* = 7042)	*M* = 3.450	*M* = 3.011	*M* = 3.002	*M* = 2.055
	SD = 1.130	SD = 0.286	SD = 0.312	SD = 0.328
Words under the label ABSTRACTION in WN (*n* = 3896)	*M* = 2.754	*M* = *M* = 2.947	*M* = 2.931	*M* = 1.944
	SD = 0.910	SD = 0.290	SD = 0.278	SD = 0.263
Words under the label PHYSICAL_ENTITY in WN (*n* = 3146)	*M* = 4.311	*M* = 3.089	*M* = 3.089	*M* = 2.192
	SD = 0.698	SD = 0.303	SD = 0.330	SD = 0.347
*t* value	81.25	20.85	21.39	33.19
*df*	7022	6142	6147	5742
*p*	< .001	< .001	< .001	< .001
Cohen’s *d*	1.920	0.504	0.517	0.807
99% CI of Cohen’s *d*	1.83, 2.01	0.44, 0.57	0.45, 0.59	0.74, 0.87

Table 2 shows that for the whole sample of words (first row), concreteness is on average slightly skewed toward the highly concrete end, the average of the first two measures corresponds to the median of the frequency distributions, while Specificity 3 is slightly skewed toward the highly generic end of the scale. In relation to the two branches ABSTRACTION and PHYSICAL_ENTITY in WN, the second and third rows of Table 2 show that these two samples encompass words designating concepts that are significantly different in concreteness (the first branch including concepts that are more abstract than the latter) and in specificity (the first branch including concepts that are more generic than the latter). In other words, the differences in both concreteness and specificity between words in the ABSTRACTION branch, and words in the PHYSICAL_ENTITY branch are statistically significant for all considered measures.

### Discussion

Words that appear under the nodes PHYSICAL_ENTITY and under the node ABSTRACTION in WN display significantly different levels of concreteness, when we compare them using the concreteness scores collected from human judgments (Brysbaert et al. 2014). In other words, the concepts designated by the words under the node PHYSICAL_ENTITY are significantly more concrete than the concepts designated by the words under the node ABSTRACTION.

Moreover, these two groups of words have also significantly different levels of specificity: The words in the branch under PHYSICAL_ENTITY are on average significantly more specific than the words in the branch under ABSTRACTION, although the differences in their averages are very small. This suggests that concepts that are more concrete are also more specific than concepts that are more abstract, which are in turn more generic.

Given the complex scenario that emerges from these two studies, showing that the relationship between concreteness and specificity is not a simple one, we decided to cross the two variables and observe in detail what type of concepts surfaces in the four quadrants of such intersection. We report our qualitative observations in Study 3.

## Study 3

**Fig. 3:**
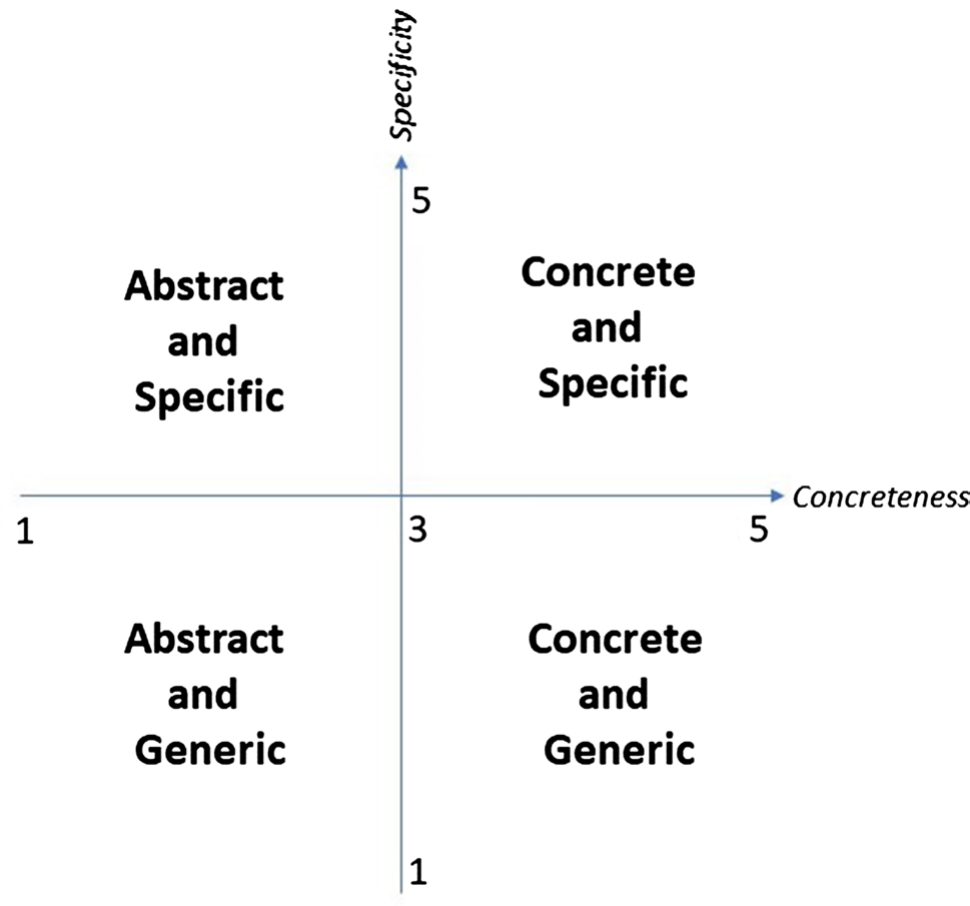
The intersection of the two variables, specificity and concreteness and the resulting four classes of concepts that we qualitatively analyzed

**Fig. 4:**
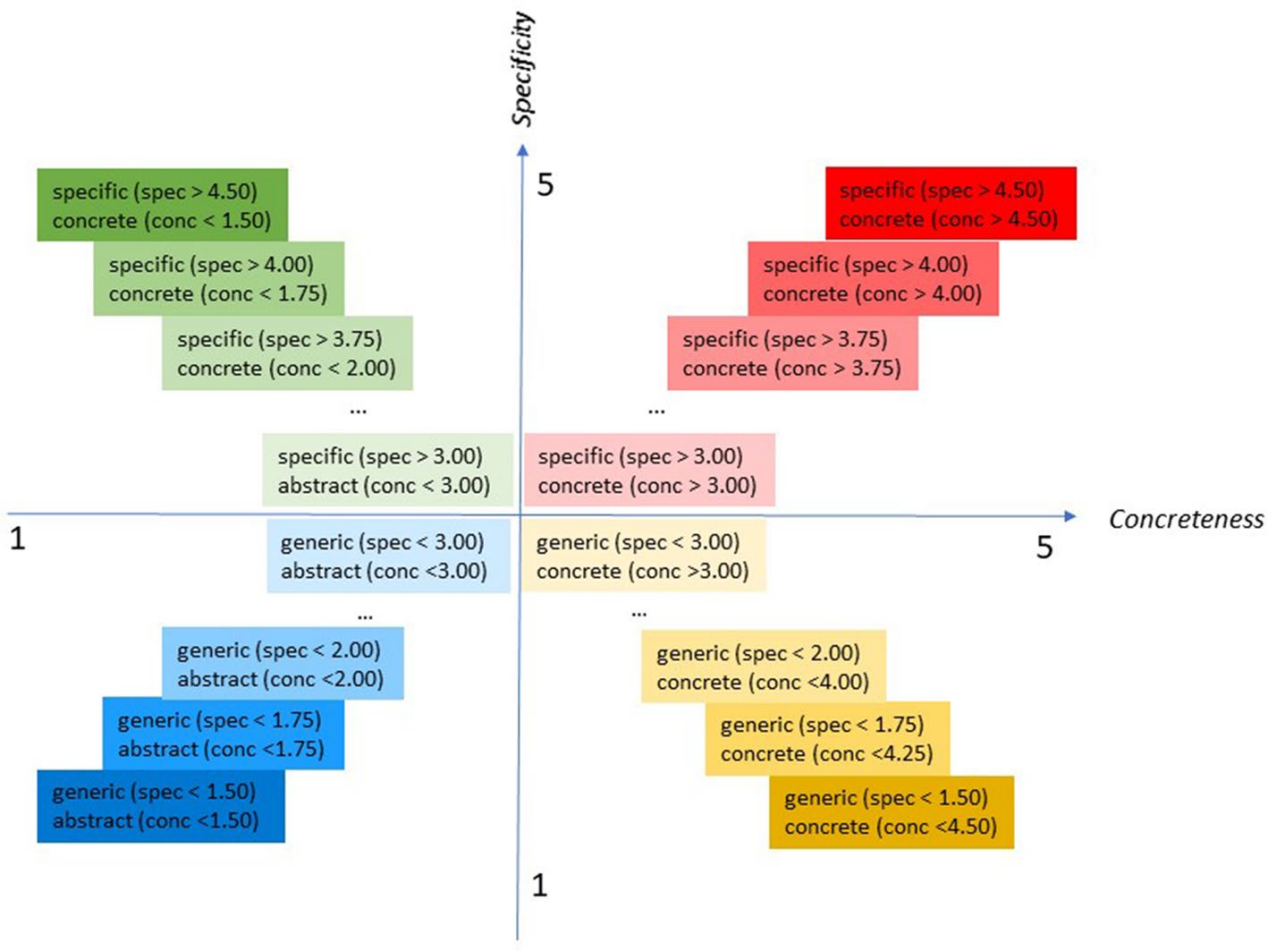
Graphic display showing how the qualitative analysis (Study 3) was implemented. The colored boxes show the ranges of concreteness and specificity scores that we extracted and analyzed, after crossing the variables concreteness and specificity

In this study, we crossed the variables specificity and concreteness, as graphically exemplified in Fig. 3, and analyzed the tails that we found by intersecting them, by gradually increasing the width of the range by 0.25 points, as graphically exemplified in Fig. 4. This gradual increase allows us to find archetypal examples of each class of concepts. Furthermore, future research could expand these classes, e.g., by considering concreteness and specificity in languages other than English.

### Materials and procedure

For each of the 13,518 words analyzed in Study 1, we considered its concreteness score and its specificity, calculated with Specificity 3, which is the measure that gave us the highest correlations with the concreteness ratings in Study 1. Moreover, as described in “Specificity measures” Section, this measure might be less skewed than Specificity 1 and Specificity 2 toward the information (i.e., number of nodes) provided by the lower part of the taxonomy (i.e., the hyponyms). Nonetheless, determining the amount of information that contributes to determine a word’s specificity in the mind of the speakers remains an open empirical question to be investigated by means of appropriate psycholinguistic tasks.

We started by selecting and analyzing the words that have scores of specificity and concreteness within the first and last 0.25 point range of the scale. For example, for the highly specific and highly concrete words we considered words displaying values of specificity > 4.75 and concreteness ratings > 4.75.

We then enlarged our range to words that have scores of specificity and concreteness within the first and last 0.5 point range of the scale. We repeated this procedure six times, by gradually enlarging the size of the tails of our words distributions by 0.25 points each time.

### Results

**Fig. 5:**
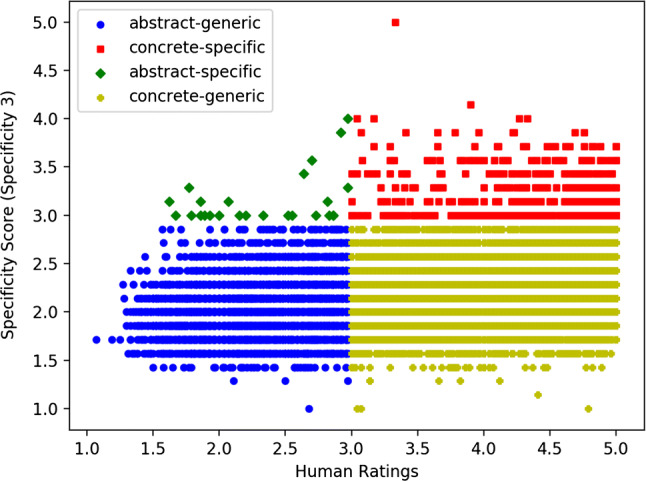
Plot showing the distribution of the 13,518 nouns across the four quadrants, obtained by crossing the variables Specificity and Concreteness

The distribution of the concreteness and specificity scores across the four quadrants is displayed in Fig. 5.

As the plots show, most of the words appear in the lower two quadrants, which display concepts that are highly abstract and generic (bottom left), and highly concrete and generic (bottom right). Concrete and specific concepts are also well represented (top right), while abstract, but specific concepts are scarcely represented (top left). Overall, it can be argued that specificity is a property that characterizes concrete concepts (which can be both, specific or generic) more than abstract concepts (which tend to be for the large majority generic).

#### Concepts that are low in specificity and low in concreteness (generic and abstract)

Overall, there are no words of this kind in the last 0.5 point above/below the minimum/maximum of the scale (specificity: 1.00 < *x* < 1.50 and concreteness: 1.00 < *x* < 1.50).

When we raised the bar by 0.25 points on both variables (specificity: 1.00 < *x* < 1.75 and concreteness: 1.00 < *x* < 1.75), 145 words emerged in the intersection of the two variables,^13^ including *absurdity*, *adaptability*, *ambience*, *ambivalence*, *amorality*, *applicability*, *aptitude*, *authenticity*, *belief*, *circumstances*, *commitment*, *contrary*, *desire*, *destiny*, and *idea*. These words appear to denote archetypal abstract concepts.

By enlarging the tails of low specificity and low concreteness by 0.25 points at the time, this tendency seems to be confirmed. As a matter of fact, words returned within the ranges of specificity: 1.00 < *x* < 2.00 and concreteness: 1.00 < *x* < 2.00 (*N* = 599) include, besides the words in the previous tails, words such as *originality*, *future*, *disparity*, *simplification*, *wrongness*, *tenacity*, *desperation*, *unpopularity*, and *criminality*.

By enlarging the tails by an additional 0.25 points, the tendency seems to remain the same. Within the ranges of specificity: 1.00 < *x* < 2.25 and concreteness: 1.00 < *x* < 2.25 (*N* = 1693), words such as *savagery*, *occultism*, *deliberation*, *ecstasy*, *cordiality*, *assertion*, and *dissemination* are added to the pool. By enlarging the tails by an additional 0.25 points (specificity: 1.00 < *x* < 2.50 and concreteness: 1.00 < *x* < 2.50 (*N* = 2627), words such as *discontinuation, plasticity, aesthetics, agony, prerequisite*, and *discomfort* are added to the pool. Once again, at first glance, all the words in this intersection express genuine abstract concepts, that is, the archetypal types of concepts used in the literature to exemplify what abstract concepts are: concepts that cannot be directly perceived through our sensory experiences, often loaded with emotional information (Vigliocco et al., 2013).

#### Concepts that are high in specificity and low in concreteness (specific and abstract)

Overall, these words seem to be not well represented, in the tails of our specificity and concreteness distributions. This goes in line with the observation provided in Table 1 and visualized in Fig. 5, according to which the specificity values are skewed toward the highly generic end, while the concreteness values are slightly skewed toward the highly concrete end (bottom quadrants in Fig. 5). Therefore, it could be expected that there are no words in the thinnest tails of highly specific and highly abstract words. We found empty intersections until we hit the range of specificity 3.25 < *x* < 5 and concreteness 1 < *x* < 2.75. At this level, the following three words emerged in the intersection of the two variables: *cakewalk, fundamentalism*, and *vintage*.

And by enlarging the range by an additional 0.25 points (specificity 3 < *x* < 5 and concreteness 1 < *x* < 3), besides the three words previously indicated, the following 7 words appeared: *bootleg*, *finisher*, *general*, *mankind*, *monotheism*, *polytheism*, and *summons*.

These words seem to be partially related to the spiritual domain and other specific human-born creations, thus identifying what is called social reality: specific creations shaped by cultural environment that emerge thanks to social interactions which is distinguished from biological or individual cognitive reality. All these words seem to express concepts that are needed to humans to categorize social and cultural experiences and talk about concepts that exist only by virtue of humans being in contact with other humans. While it can be argued that also the generic and abstract concepts denote human-born creations, it appears that, being more generic, these are less likely to be as heavily shaped by the cultural and linguistic environment as the concepts that appear in the category specific and abstract. Concepts that are generic and abstract, such as IDEA, DESIRE, and BELIEF, can be configured into different (more specific) concepts, which are in turn shaped by cultural and linguistic environment. Conversely, concepts in the category specific and abstract (e.g., CAKEWALK, which denotes a traditional American dance; BOOTLEG, which denotes a counterfeit or unofficial product, SUMMONS, which denotes an order to appear before a judge), on average appear to be notions that are already very much embedded in the cultural and linguistic environment in which they are used. This intuition suggests that concepts that are specific and abstract are less likely to be translatable into different languages, compared to concepts that are generic and abstract. For the purpose of this study, however, this remains an open empirical question to be addressed in further investigations.

#### Concepts that are high in specificity and high in concreteness (specific and concrete)

Within the last 1 point of the distributions, the intersection between highly specific and highly concrete words is empty (specificity 4 < *x* < 5 and concreteness 4 < *x* < 5).

Moving onto a slightly more encompassing intersection (specificity 3.75 < *x* < 5 and concreteness 3.75 < *x* < 5), a sample of 10 words is returned. Interestingly, this sample includes a good number of words related to chemicals and other substances: *karaoke*, *epinephrine*, *aspirin*, *heifer*, *triglyceride*, *glucose*, *chloroform*, *fructose*, and *petroleum*.

When the sample is enlarged by 0.25 points (specificity 3.50 < *x* < 5 and concreteness 3.50 < *x* < 5), a larger sample is returned (*N* = 174), belonging to a wide variety of semantic domains, including animals (*antelope*, *bison*, *cat*), food (*bread*, *grape*, *pear*), types of people (*priest*, *physician*, *parent*), and tools (*scalpel*, *scissors*, *knife*). These seem to reflect the typical categories in which we classify prototypical concrete concepts, i.e., concepts that designate tangible referents.

#### Concepts that are low in specificity and high in concreteness (generic and concrete)

There are only three words of this kind if we consider the last 0.5 point above/below the minimum/maximum of the scale (specificity 1 < *x* < 1.50, concreteness 4.50 < *x* < 5). These are *ground*, *people* and *ribbon*.

When we raised the bar by 0.25 points on both variables (specificity 1 < *x* < 1.75, concreteness 4.25 < *x* < 5), 273 words emerge from the intersection of the two variables, including: *seafood*, *ashes*, *breath*, *cloth*, *college*, *daytime*, *fabric*, and *forest*.

Many of these words, interestingly, are mass nouns, and thus denote referents that do not have clear-cut boundaries or shapes.

Finally, by raising the bar to words within the range of specificity 1 < *x* < 2 and concreteness 4 < *x* < 5, we obtain an extensive list of words (*N* = 783) belonging to several semantic domains, such as locations (*bank*, *beach*, *coast*), events (*ballgame*, *birth*, *explosion*), materials (*cashmere*, *flannel*, *plastics*), and other mass nouns (*crowd*, *herd*, *potpourri*).

### Discussion

Through a qualitative analysis of the words denoting concrete and specific, concrete and generic, abstract and concrete, and abstract and generic concepts, we observe some tendencies that we hereby summarize.

First, the prototypical abstract concepts, which are traditionally mentioned to exemplify what abstract concepts are, seem to be highly generic as well. In other words, when we refer to abstract concepts as concepts that cannot be directly perceived through our sensory experiences, because they lack a perceptible referent in the world, we refer to concepts that are also quite generic, in terms of conceptual (categorical) abstraction.

Second, there is another type of concepts that score low in concreteness, and are therefore abstract concepts, but also quite specific, in terms of categorical level of abstraction. These words, which in our study relate mainly to the spiritual domain or to the socio-political one, belong to social reality, which describes phenomena that would not exist independently of the attitudes of people toward them. Such phenomena depend on our mind to exist, but emerge thanks to social interactions with other human beings, and are therefore distinguished from both biological reality and individual cognitive reality.

Third, the prototypical concrete concepts, which are traditionally mentioned to exemplify what concrete concepts are, seem to be also highly specific. In other words, when we refer to concrete concepts as concepts that can be directly perceived through our sensory experiences because they designate tangible referents in the world, we refer to concepts that are also quite specific, in terms of categorical level of abstraction.

Fourth, there seems to be a type of concrete concepts which are also quite generic in terms of conceptual (categorical) abstraction. These concepts seem to include several mass nouns, or uncountable nouns, and therefore denote concrete referents in the world that do not have clearly defined boundaries. Interestingly, Markman (1985) offers a functional explanation as to why generic category terms as *furniture*, *jewelry*, and *money* tend to be mass nouns although they refer to classes of (diverse) concrete objects. The author explains that mass terms typically refer to mass-like relatively homogeneous substances, such as *milk* or *sand*. However, on the basis of research contrasting collective and count nouns, she argues that by using mass nouns to refer to superordinate categories (*furniture*, *jewelry*) languages could help speakers learn the hierarchical relations between superordinate and lower-level categories.

These concepts (concrete but generic) have a peculiar relation to the structure of conceptual metaphors, which we shall spell out. An important goal ascribed to concrete concepts is that they can be used as source domains to metaphorize more abstract concepts. For instance, conceptual metaphor theory (Lakoff and Johnson 1980) proposed that abstract concepts (e.g., LIFE) are typically understood metaphorically through more concrete concepts (e.g., JOURNEY). Burgers and Ahrens (in press) recently reported on a study of metaphors for the concept TRADE in a corpus of 225 years of State of the Union speeches delivered by US presidents. They found that metaphors for TRADE that drew from concepts that were concrete but generic were likeliest to be used over a long period of time. After all, these concepts were concrete and could thus be used for sense-making of a relatively abstract concept like trade. Furthermore, the genericity of these concepts also made them more adaptable to changing situational circumstances than more specific concepts. Similarly, some of the generic-concrete words found in our analysis are also used often as metaphorical source domains. The prime example is *people*, as metaphors ascribing human characteristics to non-human entities (known as “anthropomorphism” or “personification”) are often used in discourse (e.g., Dorst 2011; Epley et al. 2008).

To conclude, this preliminary qualitative exploration of the concepts that appear in the four quadrants obtained by crossing concreteness with specificity suggests that a further, more systematic empirical analysis is needed to clarify whether these four groups are substantially different from one another, and whether other differences (than abstractness and specificity) may hold between them. For example, it could be interesting to explore whether the differences between superordinate and basic-level categories reported in the literature for concrete concepts hold for abstract concepts as well.

## General discussion

In the Introduction to our study, we formulated our first research question as follows:

- RQ1: Are *generic* concepts perceived to be more abstract than *specific* ones?

In Study 1, we showed a medium–low significant correlation between the two variables of Concreteness (which measures concepts on a scale that goes from highly concrete to highly abstract) and Specificity (which measures concepts on a scale that goes from highly specific to highly generic) that can be mapped to the notions of Abstractness and Abstraction, following the terminology adopted by Borghi and Binkofski 2014. However, we also acknowledged that WN presents some problematic issues that affect the analyses of categorical specificity. Notably, some words in WN have multiple inheritances (i.e., the same word sense has multiple hypernyms) which in the hierarchy of the WN taxonomy end up on different root nodes. Among these root nodes, and therefore still at a very generic level, WN distinguishes between ABSTRACTION and PHYSICAL_ENTITY, which seems to be a distinction highly connected to our variable Concreteness. Some words appear within both of these branches, because they have multiple hypernyms that follow different routes to the top of the WN taxonomy. This suggests that even though we used the same word sense for these words (as listed in WN), these words still conflate different meanings, and designate different referents, which in some cases differ in terms of concreteness. Reijnierse et al. (2019) claim that this specific type of polysemy, for which a meaning is significantly more concrete than another, is peculiar of metaphoricity and characterize words that have a literal meaning (typically more concrete, e.g., *support*: physical tool like a wooden board) and a derived, metaphorical one (typically more abstract, e.g., *support*: psychological help).

Our second RQ was formulated as follows:

- RQ2: To which extent does the distinction between abstract and concrete entities in the WordNet (WN, henceforth) taxonomy (Fellbaum 1998) reflect the concreteness perceived by speakers? And if the WN-based distinction between abstract and concrete is backed up by participants’ judgments, do the words within these two WN branches display statistically significant differences in their average degree of specificity?

In Study 2, we recast our analyses. On the one hand, we exploited the distinction between physical and abstract entities featured in WN and, on the other hand, we excluded all words that have multiple inheritance. We then tested whether the words within each of these two branches of the WN taxonomy designate concepts that are, on average, significantly different in terms of concreteness and in terms of specificity. We found that words that appear in the WN branch under the node ABSTRACTION are significantly less concrete and less specific than the words that appear in the WN branch under the node PHYSICAL_ENTITY. The correlation coefficients between specificity and concreteness within each of the two branches increased, compared to the results obtained in Study 1.

Finally, our third RQ was formulated as follows:

- RQ3: What type of concepts can be found in the four intersections of the two variables, specificity and concreteness?

In Study 3, we provided a qualitative analysis of the words that appear to be highly concrete and highly specific, highly concrete and highly generic, highly abstract and highly specific, and highly abstract and highly generic. We observed that while abstract and generic words designate prototypical abstract concepts, which are likely to be loaded with emotional information, abstract and specific words designate specific concepts within the social reality domains. Moreover, while concrete and specific words designate prototypical concrete concepts, belonging to several domains such as animals, food, and tools, concrete and generic concepts appear to include a variety of mass nouns, and to be in general good candidates as source domains in metaphorical comparisons.

This study aimed at disentangling and clarifying the relation between specificity and concreteness, two variables that get often conflated when referring to the general phenomenon of abstraction. We showed that such relationship is far from being trivial. Specificity and concreteness appear to be mildly correlated: They do not go hand in hand. Crossing the two variables, we obtained words that seem to designate qualitatively different concepts, in the four derived quadrants.

Within the cognitive sciences, our findings can be interpreted as follows. Starting from the buzzy world of perceptual experiences, on the one hand we have the construction of conceptual categories that vary in terms of granularity (categorical abstraction from specific to generic). On the other hand, we have the construction of conceptual representations, some referring to what can be directly perceived through perceptual experiences, some referring to intangible entities (conceptual representation of abstractness/concreteness). In this sense, the two variables, specificity and concreteness, might identify two phenomena that differ in the way concepts get access to the designated referents in the world. In particular, specificity might characterize the way in which concepts get access to the referents via language, while concreteness might characterize the way in which concepts get access to the referents via mental representations. Moreover, specificity is measured as a characteristic (which we operationalized through WN), while concreteness is measured as a perception variable (which is commonly operationalized through concreteness ratings). Therefore, they constitute two distinct types of variables as well.

Some caveats need to be discussed, in relation to our findings, which are based on WN and the concreteness ratings paradigm. First, WN is a lexicon that has been used in a variety of studies and works. Nevertheless, this resource has been created and conceived with some psycholinguistic assumptions, especially as far as its taxonomy is concerned (see Miller 1998 for a detailed illustration of the modeling principles of the noun taxonomy). This can have an impact in the results that may be obtained when applied in other contexts. For instance, multiple inheritance in WN is presented and justified as way of modeling different meanings of the hyponym/hypernym relations (Miller 1998). Thus, multiple inheritance in WN seems very useful if someone wants to study the continuum between abstract and concrete nouns and investigate whether some modeling decisions are reflected by human judgments. For our study, multiple inheritance, especially when it involves incompatible ontological categories, is a source of noise and breaks some assumptions related to the use of taxonomies to represent nominal meanings.

Second, the WN noun taxonomy and its sense repository (synsets) are very fine-grained. Some studies have criticized this fine grained-ness representation, especially for applicative goals (Weischedel et al. 2011). In addition to this, senses in WN do not have any specific order. However, as an effort to include more contextual information in WN, there have been initiatives on manually annotating corpora with WN synsets (Miller et al. 1993, Ide, 2012, among others). This has resulted in the possibility of “re-ordering” some senses in WN according to their frequencies in these annotated corpora. Nevertheless, the texts composing these corpora may have biased the ordering of the senses. For instance, the noun *tablespoon* is associated with a synset composed of the synsets words “tablespoon, tablespoonful.” The noun *tablespoonful* has been annotated in the SemCor Corpus (Miller et al. 1993) with the meaning of “as much as a tablespoon will hold.” On the other hand, the meaning “a spoon larger than a dessert spoon; used for serving” of *tablespoon* has never been annotated. It follows that the first sense of *tablespoon* in WN is “as much as a tablespoon will hold,” and such sense belongs to the branch under the node ABSTRACTION.

Finally, in relation to the concreteness ratings that we used (Brysbaert et al. 2014) it shall be mentioned that these types of ratings, largely used in cognitive science and cognitive psychology to operationalize conceptual concreteness, are typically collected by presenting words to participants, without providing any context that could help the participants disambiguating potential polysemic words. Therefore, participants submitted to rate the concreteness of, for example, *support*, as described above, may in principle rate either the concrete or the abstract (figurative) sense of this word, especially given the fact that they are both frequently used word senses. This problem relates to the multiple inheritance problem that characterizes WN.

## Conclusion and outlook

In this work, we have investigated the relationship between concreteness and specificity looking for empirical evidence that may support the hypothesis that generic categories (i.e., low in specificity) are less rich in perceptual features, and therefore more abstract. Results seem to point out that these two variables capture theoretically distinct concepts, that can be crossed and give rise to sub-types of words. If such distinction of concreteness and specificity as independent theoretical notions is accepted, as a consequence the construct of “abstraction” needs to be revised as well.

*Abstraction* is a polysemic word that can be used to define the extraction of information from perceptual experiences to construct and label with words semantically coherent categories at various levels of granularity (e.g., TABLE, FURNITURE, ENTITY), as well as the construction of mental representations that group together various experiences on the basis of some type of perceived similarity between them (e.g., TABLE, DEBATE, JUSTICE). These two meanings refer to what in cognitive science is usually referred to as the difference between a concept (a mental representation of classes of things) and a category (referring the labeled classes themselves) (e.g., Murphy 2002), although such distinction is not free from critiques.

Consider the three pillars around which much of the cognitive scientific work revolves, and the mutual relations between them: thought, language, and world. The process of categorical abstraction thanks to which we construct the category TABLE from individual instances that we can directly experience (e.g., your kitchen table) and the category FURNITURE by grouping together tables, chairs and couches, is a process in which *language* allows us to glue together individual experiences—and label them with a word. The variable that we called specificity therefore describes how perceptual experiences derived from the world are categorized *by language*. Conversely, conceptual concreteness describes how perceptual experiences derived from the world are categorized *by thought*, to construct mental representations. If this is the case, abstraction needs to be investigated in a cross-disciplinary manner: Linguists, communication scientists, psychologists, cognitive scientists, and computer scientists need to be working together, use a shared terminology, and apply a variety of methods to shed lights on such a complex phenomenon.

## Footnotes

1 The difference between concepts and categories has been debated for decades within the cognitive-science and psychology communities. We refer here to concepts as mentally possessed ideas or notions, and to categories as sets of entities that are grouped together (as, for example, in Barsalou 1983; Goldstone and Kersten 2003; Goldstone et al. 2019). In this sense, the concept TABLE is whatever psychological state signifies thoughts of tables. The category TABLE consists of all the entities in the real world that are appropriately categorized as tables. Please see the Discussion section for further elaboration on this matter.

2 We specify ‘directly’ because various theoretical accounts, expressed especially within the grounded cognition framework (e.g., Barsalou 2008), suggest that abstract concepts can in fact be grounded in perception (and action, and emotion) in various indirect ways (see Bolognesi and Steen (2019) for an extensive and recent debate on this topic).

3 These two variables are defined as such by Borghi and Binkofski (2014), and discussed extensively in Bolognesi and Steen (2019).

4 All the materials related to our analyses are stored online on Open Science Framework (OSF): https://bit.ly/2TWROm7.

5 Definition number 3 relates to conditioning and defines abstraction as “discrimination based on a single property of multicomponent stimuli.” For the purpose of our argumentation, we are not going to discuss this third definition further.

6 We used version 3.2.2. of NLTK.

7 We have included also IS-INSTANCE relations.

8 Concepts represented by synsets words that do not appear in such semantically annotated resources are ordered randomly.

9 Iliev and Axelrod (2017) took the average values of the inclusiveness scores for each synset node associated with a target synset word in case of polysemous entries. This means, for instance, that in the case of the word “dog” (dog.n.01), that has two hypernyms nodes “domestic animal” and “canine,” they followed the path of “canine,” as it is the most informative (i.e., the longest). Moreover, because the synset word “dog” is polysemous (it is associated with seven different synsets), their inclusiveness score is given by the average of the inclusiveness score of each synset. We, however, consider misleading taking the average of the senses, for each concept, because the different senses may be at very different positions in the taxonomy, thus resulting in biased scores.

10 The main difference between Specificity 1 (and Specificity 2, which is a derived version) and Specificity 3, which we developed, is the following. Specificity 1 takes into account: the number of nodes to the top root node (hypernyms), the total number of hyponyms, until they reach the leaves, and the total number of nodes in the whole WordNet taxonomy. This implies that the specificity measure for the term *dog*, for example, is determined by nodes like *canine*, *carnivore*, *mammal*, *vertebrate*, *animal*, and *organism* (hypernyms) as much as by (dozens and dozens of) hyponymic nodes like *puppy*, *poach*, *cur*, *feist*, *lap-dog*, *Chihuahua*, *Japanese spaniel*, *Maltese dog*, *Pekinese*, *hound*, *Afghan hound*, *basset*, and so forth. We suspect that such measure may give too much weight to the number of (direct as well as indirect) hyponyms that constitute the lower (i.e., more specific) part of the taxonomy, from a node like *dog* to each and every possible leaf node that it may have in the taxonomy. Thus, we formulate Specificity 3, which considers the distance from a node to its top root node, and the total depth of the taxonomy (20 nodes) rather than the number of nodes in the whole WordNet taxonomy and the highly variable number of nodes existing under each node (e.g., from *dog* to any of its leaves, all summed up together).

11 We also verified this in a regression model: When entering all three specificity measures in a regression simultaneously, we received red flags on multicollinearity, especially in relation with Specificity 1 and Specificity 2, which had very high loadings on the same eigenvalue (Specificity 1: 0.988; Specificity 2: 0.974; VIF Specificity 1: 50.484; VIF Specificity 2: 44.023). Subsequently, we estimated a regression model with only Specificity 1 and Specificity 3 as predictors of concreteness (and Specificity 2 left out). In this case, the VIF dropped considerably to 3.541 which suggests that the regression may still be biased (Field, 2013, p. 325). Furthermore, both specificity measures still have very high loadings on the same eigenvalue (Specificity 1: 1.00; Specificity 3: 0.717), which further suggests multicollinearity. A regression model with Specificity 2 and Specificity 3 as predictors showed similar results: VIF = 3.09, but both measures had very high loadings on the same eigenvalue (Specificity 2: 1.00; Specificity 3:.69).

12 We have explicitly excluded the other top node, THING, for the following reasons: It only contains 8 hyponym synsets, all at distance 1; the set of synsets under THING is vague and may be used to refer to both physical and abstract concepts.

13 The complete list of nouns for each combination of concreteness and specificity is available in the linked OSF repository.
